# Supplementary material for: Inhibitory Effect of pH-Responsive Nanogel Encapsulating Ginsenoside CK against Lung Cancer
Source: Polymers (Basel). 2021 May 28;13(11):1784. doi: 10.3390/polym13111784 (PMC8198720; doi:10.3390/polym13111784)
Supplement: Supplementary file 1 [file polymers-13-01784-s001.zip › polymers-1203234-supplementary.pdf]

Inhibitory Effect of pH-Responsive Nanogel Encapsulating Ginsenoside CK against Lung Cancer

Ziyang Xue [1,2,†], Rongzhan Fu [1,2,†], Zhiguang Duan [1,2], Lei Chi [1,2], Chenhui Zhu [1,2,*] and Daidi Fan [1,2,*]

[1] Shaanxi Key Laboratory of Degradable Biomedical Materials and Shaanxi R&D Center of Biomaterials and Fermentation Engineering, School of Chemical Engineering, Northwest University, Taibai North Road 229, Xi'an, Shaanxi, 710069, China

[2] Biotech & Biomed Research Institute, Northwest University, Taibai North Road 229, Xi'an, Shaanxi, 710069, China

† These authors contributed equally to this work.

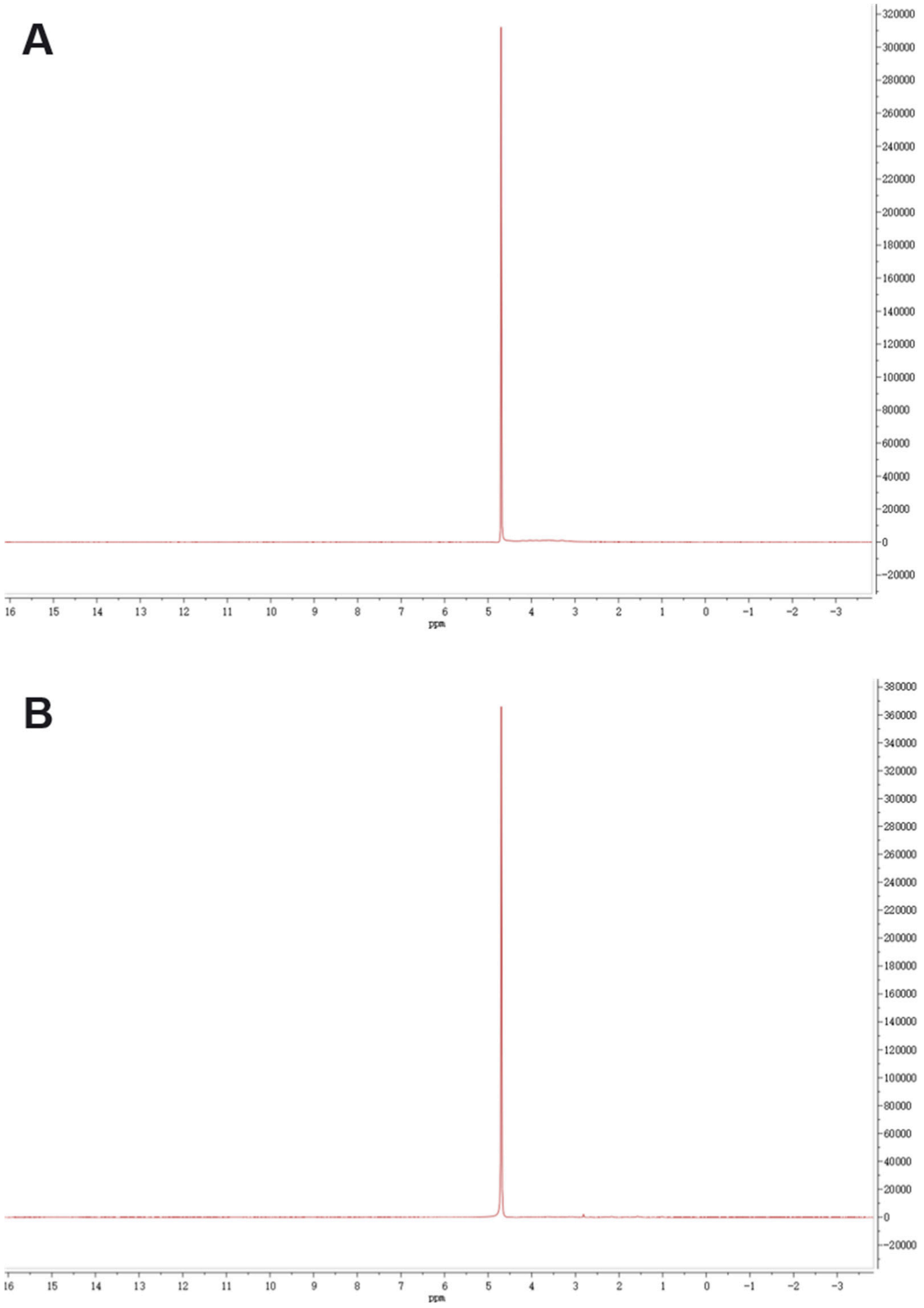

Figure S1. ^{1}H NMR raw spectrum of CMC (**A**), and CMC-NH_2 (**B**).
